# The involvement and therapeutic potential of lncRNA Kcnq1ot1/miR-34a-5p/Sirt1 pathway in arsenic trioxide-induced cardiotoxicity

**DOI:** 10.1186/s12967-023-03895-0

**Published:** 2023-01-28

**Authors:** Xiuyun Shen, Fengnan Zhi, Chunpeng Shi, Jincheng Xu, Yuqiu Chao, Juan Xu, Yunlong Bai, Yanan Jiang, Baofeng Yang

**Affiliations:** 1 Department of Pharmacology (State-Province Key Laboratories of Biomedicine-Pharmaceutics of China, Key Laboratory of Cardiovascular Research, Ministry of Education), College of Pharmacy, Harbin Medical University, Harbin, China; 2 Translational Medicine Research and Cooperation Center of Northern China, Heilongjiang Academy of Medical Sciences, Harbin, China; 3 College of Bioinformatics Science and Technology, Harbin Medical University, Harbin, China; 4 Research Unit of Noninfectious Chronic Diseases in Frigid Zone, Chinese Academy of Medical Sciences (2019RU070), Harbin, China

**Keywords:** Arsenic trioxide, Cardiotoxicity, LncRNA Kncq1ot1, Propranolol

## Abstract

**Background/Aims:**

Arsenic trioxide (ATO) is the first-line therapeutic drug for acute promyelocytic leukemia. However, the cardiotoxicity of ATO limits its clinical application. This study aims to explore the long noncoding RNA (lncRNA) involved molecular mechanism in ATO-induced cardiotoxicity and to identify available prevention strategies.

**Methods:**

ATO was administered to mice or primary cultured mouse cardiomyocytes. Small interfering RNA targeting lncRNA Kcnq1ot1 (si-Kcnq1ot1) was used to knockdown lncRNA Kcnq1ot1. MiR-34a-5p mimic and antisense morpholino oligonucleotide targeting miR-34a-5p (AMO-34a-5p) were used to upregulate and downregulate the expression of miR-34a-5p, respectively. TUNEL staining was conducted to detect cell DNA damage. Flow cytometry assay was used to detect cell apoptosis. Western blot was conducted to detect Bcl-2, Bax and Sirt1 protein expression. Real-time PCR was used to detect lncRNA Kcnq1ot1, miR-34a-5p, and Sirt1 mRNA expression. Dual-luciferase reporter assay was performed to validate the predicted binding site.

**Results:**

ATO induced apoptosis in cardiomyocytes both in vivo and in vitro. Simultaneously, the expression of lncRNA Kcnq1ot1 and Sirt1 was downregulated, and miR-34a-5p was upregulated. MiR-34a-5p has binding sites with lncRNA Kcnq1ot1 and Sirt1. Knockdown of lncRNA Kcnq1ot1 induced apoptosis of cardiomyocytes, with increased miR-34a-5p and decreased Sirt1 expression. Inhibition of miR-34a-5p attenuated si-Kcnq1ot1-induced apoptosis in cardiomyocytes. Therefore, the lncRNA Kcnq1ot1/miR-34a-5p/Sirt1 signaling pathway is involved in ATO-induced cardiotoxicity. Propranolol alleviated ATO-induced apoptosis in cardiomyocytes both in vivo and in vitro, which was related to the lncRNA Kcnq1ot1/miR-34a-5p/Sirt1 signaling pathway.

**Conclusion:**

The lncRNA Kcnq1ot1/miR-34a-5p/Sirt1 pathway is involved in ATO-induced cardiotoxicity. Propranolol can attenuate ATO-induced cardiotoxicity at least partially through the lncRNA Kcnq1ot1/miR-34a-5p/Sirt1 pathway. Combined administration with propranolol may be a new strategy for alleviating the cardiotoxicity of ATO.

## Introduction

Arsenic trioxide (ATO) is an important clinical therapeutic drug for leukemia [1] and liver cancer [2, 3], which also has therapeutic potential for breast cancer [4, 5], lung cancer [6, 7], and gastric cancer [8, 9], etc. However, ATO can induce some toxic or side effects, including cardiotoxicity [10, 11], liver toxicity [12], and kidney toxicity [13]. Among them, cardiotoxicity is the main reason that limited the clinical use of ATO [14, 15]. However, the mechanism underlying ATO-induced cardiotoxicity has not been fully elucidated.

Noncoding RNAs, such as microRNAs (miRNAs) and long noncoding RNAs (lncRNAs), play pivotal roles in various cardiac diseases, such as cardiac hypertrophy, myocardial infarction, and heart failure [16–18]. MiRNAs can bind to the 3’UTR of target genes, thus regulating gene expression. LncRNAs can exert gene regulatory functions in different ways. In the competing endogenous RNA (ceRNA) mechanism, noncoding RNAs (such as lncRNAs) can interact with miRNAs, thus regulating target mRNA expression. This mechanism is considered the Rosetta Stone of a hidden RNA language [19]. Many lncRNAs are involved in cardiac apoptosis through competitive binding with miRNAs [20]. For example, the lncRNA MIRF contributes to cardiac apoptosis through regulation of the miR-26a-Bak1 signaling pathway [21].

One of the major mechanisms of ATO-induced cardiotoxicity is induction of apoptosis [22–24]. Noncoding RNAs are also involved in ATO-induced cardiotoxicity. LncRNA NEAT1 was found to be downregulated in ATO-treated H9c2 cardiomyocytes. Enhanced expression of lncRNA NEAT1 protected H9c2 cardiomyocytes against ATO-induced injury through the miR-124/NF-κB signaling pathway [25]. Our previous study demonstrated that ATO-induced QT interval prolongation of electrocardiograms (ECGs) was related to inhibition of lncRNA Kcnq1ot1 [26]. In addition, recent studies verified that lncRNA Kcnq1ot1 contributes to the apoptosis process [27, 28]. However, the involvement of lncRNA Kcnq1ot1 in ATO-induced apoptosis of cardiomyocytes remains unclear. Therefore, based on our previous work and the existing findings, the present study aims to clarify the underlying mechanism and to explore the therapeutic potential of lncRNA Kcnq1ot1 in ATO-induced cardiomyocytes apoptosis.

## Materials and methods

### Animals and treatment

C57BL/6 mice (20–22 g) were obtained from Liaoning Changsheng Biotechnology Co., Ltd. (China). The experimental procedure was approved by the Experimental Animal Ethics Committee of Harbin Medical University, China (No. HMUIRB 20150034). Mice were administered ATO (1.5 mg/kg/day, intraperitoneal injection; Harbin Yida Pharmaceutical Co., Ltd., China) alone or in combination with propranolol (10 mg/kg/day, intragastric administration; YABANG Pharma, China) for 2 weeks.

### Primary culture of neonatal mouse cardiomyocytes

Cardiomyocytes were isolated from neonatal mice (1 to 3 days). Myocardial tissues were digested with 0.25% pancreatin (Solarbio, China). After filtering and centrifugation (1500 revolutions per minute at 4 °C for 5 min), the isolated cells were collected and cultured in Dulbecco’s modified Eagle’s medium (DMEM; HyClone, USA) with 10% fetal bovine serum (FBS; BI, Israel) for 1.5 h to remove noncardiomyocytes. Cardiomyocytes were seeded into another culture plate and incubated under 5% CO_2_ at 37 °C [26, 29, 30]. After 48 h, the cardiomyocytes were used for the following experiments.

### Treatment and transfection of neonatal mouse cardiomyocytes

ATO (5 μM, Harbin Yida Pharmaceutical Co., Ltd., China) and propranolol (10 μM; Sigma, USA) were added to the culture medium for 48 h. MiR-34a-5p mimic, antisense morpholino oligonucleotide targeting miR-34a-5p (AMO-34a-5p) and the corresponding negative controls (miR-NC and AMO-NC) were biosynthesized by RIBOBIO (China), and the working concentration was 5 nmol/250 μL. Small interfering RNA targeting lncRNA Kcnq1ot1 (si-Kcnq1ot1) and the negative control (si-NC) were biosynthesized by GenePharma (Shanghai, China), and the working concentration was 1 OD_260_/125 μL [26]. For the 12-well plate system, each well required 50 μL Opti-MEM (Gibco, USA), 3 μL X-tremeGENE siRNA Transfection Reagent (Roche, Switzerland) and 9 μL miRNA/siRNA. For the 96-well plate system, each well required 20 μL Opti-MEM, 0.5 μL X-tremeGENE siRNA Transfection Reagent and 1.5 μL miRNA/siRNA. After transfection, the culture plates were placed in an incubator containing 5% CO_2_ at 37 °C for 48 h.

### CCK-8 assay

Cardiomyocytes were cultured in a 96-well plate at a density of 2.5 × 10^4^ cells/well. After treatment, the culture medium of cardiomyocytes was removed, and CCK-8 (DOJINDO, China) solution was added. The plate was incubated at 37 °C for 2.5 h. The OD value of each well was measured at 450 nm. The calculation formulas were as follows: Cell viability (%) = (experiment group−blank well)/(control group−blank well) × 100% [31]; Inhibition rate (%) = (control group−experimental group)/(control group−blank well) × 100% [32].

### TUNEL assay

Briefly, 5 µm frozen heart tissue slides or cultured cardiomyocytes were fixed in 4% paraformaldehyde for 10 min and then washed with phosphate buffered saline (PBS; HyClone, USA) thrice for 5 min each time, followed by incubation with 0.1% Triton X-100 for 2 min and washing with PBS for 5 min. The slides were blocked with goat serum for 20 min and washed with PBS for 10 min, and TUNEL reaction mixture (50 μL; Roche, Switzerland) was added. The slides were incubated at 37 °C for 1 h, washed with PBS for 15 min, incubated with DAPI for 5 min, and washed with PBS for 10 min. A fluorescence microscope (BX53F; OLYMPUS, China) was used to capture images.

### Prediction of miRNA targets

Physical interactions between lncRNAs and miRNAs, and between miRNAs and mRNAs were considered to predict the mechanism of lncRNA Kcnq1ot1. MiRNA targets were predicted using RNAhybrid [33], Miranda [34], MIREAP[35], TargetScan [36] and ENCORI [37].

### Western blot assay

Total protein was extracted from myocardial tissues and cardiomyocytes [38]. The protein content in each lane of the same membrane is the same. Each lane was loaded with about 30–50 μg cell protein or about 80–120 μg tissues protein. The protein samples were separated via 10% SDS-PAGE, transferred to an NC membrane (Pall Corporation, USA), and incubated with primary antibodies, including antibodies targeting GAPDH (ZSGB-BIO, China), Bcl-2 (ABclonal, China), Bax (ABclonal, China) and Sirt1 (Abcam, Britain), at 4 °C overnight. Afterward, the membrane was washed and incubated with secondary antibody for 1 h at room temperature. An Odyssey infrared fluorescence scanning imaging system was used to obtain the images. The densitometry of the protein bands was quantified using Image Studio software. GAPDH served as an internal control to normalize protein expression levels. The data were normalized to the control group data.

### Real-time PCR assay

Total RNA was extracted from myocardial tissues and cardiomyocytes using RNAiso Plus (Takara, Japan). A NanoDrop spectrophotometer (Thermo Fisher Scientific, USA) was used to detect the RNA concentration and the A260/A280 ratio of the samples. An ABI 7500 Fast Real-time PCR System (ABI, USA) was used to perform qRT-PCR analysis with SYBR Green I (Toyobo, Japan) [38]. The primer sequences were shown in Table 1. U6 and GAPDH served as internal controls for miRNA and mRNA/lncRNA, respectively. The relative gene expression level was analyzed using the 2^−ΔΔCT^ method.

**Table 1:** Primer sequences

Gene name	Primer sequences
Kcnq1ot1	Forward: 5’-GCACTCTGGGTCCTGTTCTC-3’ Reverse: 5’-CACTTCCCTGCCTCCTACAC-3’
Sirt1	Forward: 5’-GACGCTGTGGCAGATTGTT-3’ Reverse: 5’-GCAAGGCGAGCATAGATACC-3’
GAPDH	Forward: 5’-AAGAAGGTGGTGAAGCAGGC-3’ Reverse: 5’-TCCACCACCCTGTTGCTGTA-3’
miR-34a-5p	Forward: 5’-GTGGCAGTGTCTTAGCTG-3’ Reverse: 5’-TATCCAGTGCGTGTCGTG-3’ Reverse transcription: 5’-GTCGTATCCAGTGCGTGTCGTGGAGTCGGCAATTGCACTGGATACGACACAACC-3’
U6	Forward: 5’-GCTTCGGCAGCACATATACTAAAAT -3’ Reverse: 5’-CGCTTCACGAATTTGCGTGTCAT-3’ Reverse transcription: 5’-CGCTTCACGAATTTGCGTGTCAT -3’

### Dual-luciferase reporter assay

HEK-293 cells were seeded in 24-well plates. Transfection was performed using Cellfectin II Reagent (Invitrogen, CA, USA) when the confluence of the cells was approximately 50%−60%. The wild-type (WT) sequence of Kcnq1ot1 is “actgcca”, while the mutation sequence was “ctgattc”. The luciferase density was detected using a Dual-Luciferase Reporter Assay System (GloMax™ 20/20; Promega, WI, USA).

### Flow cytometry assay

The cells were digested using trypsin without EDTA, washed with PBS, and resuspended in 1 × binding buffer at a concentration of 2 × 10^6^ cells/mL. The cells were stained using an Annexin V-FITC/PI Apoptosis Detection Kit (4A BIOTECH, Beijing, China) according to the manufacturer’s instructions and detected using a BD FACSCelesta™ flow cytometer. The proportion of apoptotic cells (Annexin V ( +) PI (−)) was analyzed.

### Statistical analysis

Data are presented as the mean ± SEM. Each experiment was duplicated at least three times. Comparisons between two groups were analyzed using Student’s t test; and comparisons among three or more groups were analyzed via one-way ANOVA followed by Newman-Keuls multiple comparisons. Statistical significance was defined as P < 0.05.

## Results

### ATO induced apoptosis in mouse myocardial tissues and cardiomyocytes

**Fig. 1:**
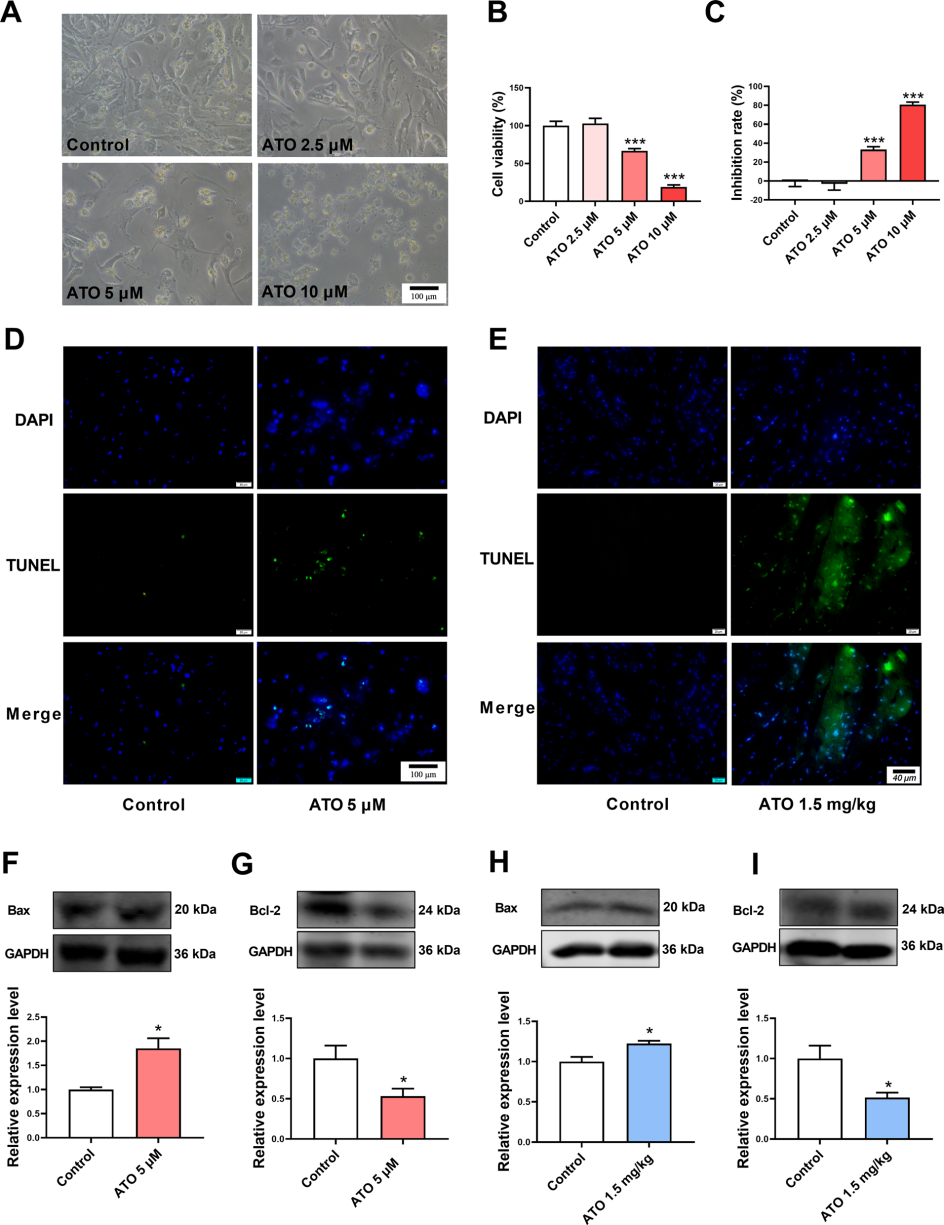
ATO induced apoptosis of cardiomyocytes both in vitro and in vivo. **A** Representative mouse cardiomyocyte images. Magnification: 200 × ; scale bar: 100 μm. **B** ATO decreased the viability of mouse cardiomyocytes. **C** ATO increased the inhibition rate of mouse cardiomyocytes. **D** Representative TUNEL staining images of mouse cardiomyocytes. Magnification: 200 × ; scale bar: 100 μm. **E** Representative TUNEL staining images of mouse myocardial tissues. Magnification: 400 × ; scale bar: 40 μm. **F** ATO increased Bax protein expression in mouse cardiomyocytes. **G** ATO decreased Bcl-2 protein expression in mouse cardiomyocytes. **H** ATO increased Bax protein expression in mouse myocardial tissues. **I** ATO decreased Bcl-2 protein expression in mouse myocardial tissues. For **B**, **C**, one-way ANOVA F value = 63.49 and 63.49, respectively. *P < 0.05, ***P < 0.001 vs. control group ; n = 3–4

CCK-8 assay results showed that 5 μM and 10 μM ATO reduced the cell viability and increased the inhibition rate of cardiomyocytes (Fig. 1A−C). The relatively low and effective dose of ATO (5 μM) was selected for further experiments. ATO induced apoptosis of cardiomyocytes, which presented as an increased number of TUNEL-positive cells (Fig. 1D), increased Bax protein expression, and decreased Bcl-2 protein expression (Fig. 1F, G).

The effect of ATO on cardiomyocytes was validated in vivo. ATO increased the number of TUNEL-positive cells in mouse myocardial tissues (Fig. 1E). Simultaneously, similar Bax and Bcl-2 expression changes were observed in ATO-treated mouse myocardial tissues (Fig. 1H, I).

### The effect of ATO on lncRNA Kcnq1ot1, miR-34a-5p, and Sirt1 expression in mouse myocardial tissues and cardiomyocytes

**Fig. 2:**
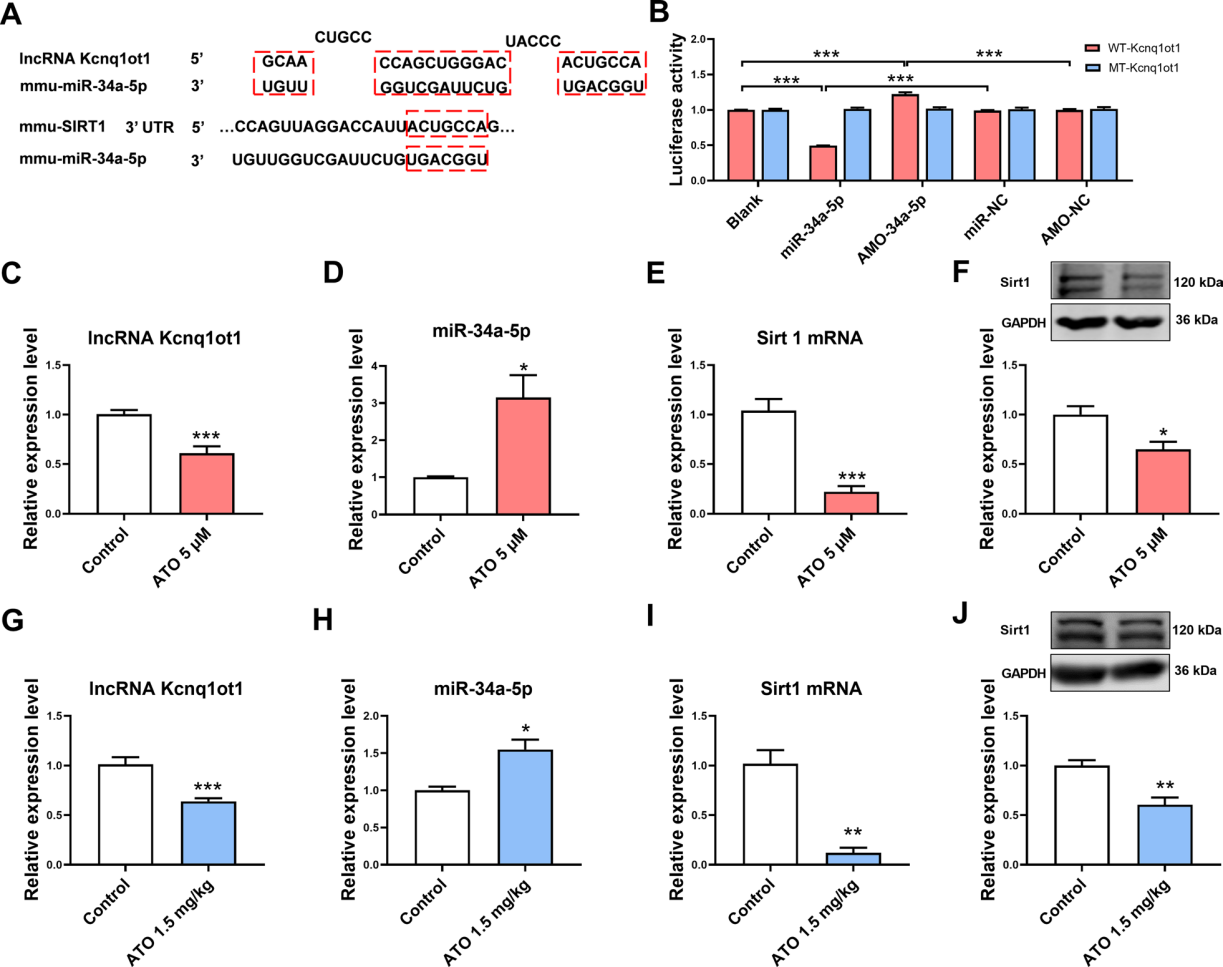
The effect of ATO on lncRNA Kcnq1ot1, miR-34a-5p, and Sirt1 expression in mouse cardiomyocytes and myocardial tissues. **A** Predicted binding sites between miR-34a-5p and lncRNA Kcnq1ot1/Sirt1. **B** The direct binding site between miR-34a-5p and lncRNA Kcnq1ot1 was validated with dual-luciferase reporter assay. **C** ATO decreased lncRNA Kcnq1ot1 expression in mouse cardiomyocytes. **D** ATO increased miR-34a-5p expression in mouse cardiomyocytes. **E** ATO decreased Sirt1 mRNA expression in mouse cardiomyocytes. **F** ATO decreased Sirt1 protein expression in mouse cardiomyocytes. **G** ATO decreased lncRNA Kcnq1ot1 expression in mouse myocardial tissues. **H** ATO increased miR-34a-5p expression in mouse myocardial tissues. **I** ATO decreased Sirt1 mRNA expression in mouse myocardial tissues. **J** ATO decreased Sirt1 protein expression in mouse myocardial tissues. For **B**, one-way ANOVA F value = 362.2. ***P < 0.001. For **C**–**J**, *P < 0.05, **P < 0.01, ***P < 0.001 vs. control group; n = 3–6

We then explored the mechanism of ATO-induced apoptosis. Based on the ceRNA theory, the target of lncRNA Kcnq1ot1 was predicted. LncRNA Kcnq1ot1 could bind with miR-34a-5p. Moreover, Sirt1 is a downstream target of miR-34a-5p (Fig. 2A). Dual-luciferase reporter assay showed that miR-34a-5p has a direct binding site with lncRNA Kcnq1ot1 (Fig. 2B). The expression levels of lncRNA Kcnq1ot1, miR-34a-5p, and Sirt1 were measured in ATO-treated mouse cardiomyocytes and myocardial tissues. The results showed that lncRNA Kcnq1ot1 and Sirt1 expression were downregulated and miR-34a-5p expression was upregulated in ATO-treated mouse cardiomyocytes (Fig. 2C–F). Similar results were observed in ATO-treated mouse myocardial tissues (Fig. 2G–J).

### The effect of miR-34a-5p on Sirt1 expression in mouse cardiomyocytes

**Fig. 3:**
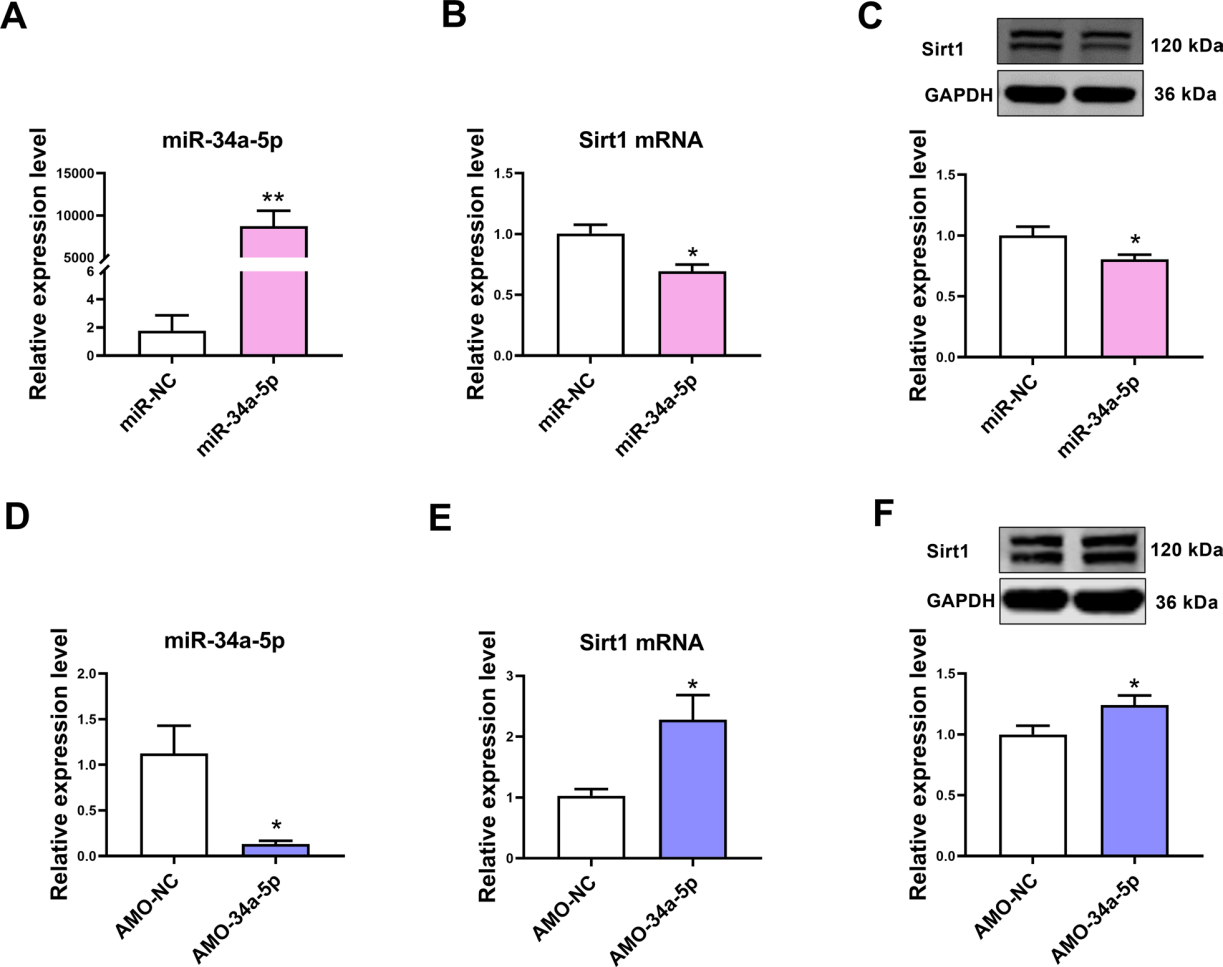
The effect of miR-34a-5p on Sirt1 expression in mouse cardiomyocytes. **A** The expression of miR-34a-5p in cardiomyocytes transfected with miR-34a-5p mimic. **B** Upregulation of miR-34a-5p inhibited Sirt1 mRNA expression in mouse cardiomyocytes. **C** Upregulation of miR-34a-5p inhibited Sirt1 protein expression in mouse cardiomyocytes. **D** The expression of miR-34a-5p in cardiomyocytes transfected with AMO-34a-5p. **E** Downregulation of miR-34a-5p increased Sirt1 mRNA expression in mouse cardiomyocytes. **F** Downregulation of miR-34a-5p increased Sirt1 protein expression in mouse cardiomyocytes. For **A**–**C**, *P < 0.05, **P < 0.01 vs. miR-NC group. For **D**–**F**, *P < 0.05 vs. AMO-NC group; n = 3–6

MiR-34a-5p expression was elevated in cardiomyocytes transfected with miR-34a-5p mimic (Fig. 3A). Overexpression of miR-34a-5p downregulated Sirt1 expression in cardiomyocytes (Fig. 3B, C). AMO-34a-5p transfection inhibited miR-34a-5p and increased Sirt1 expression in cardiomyocytes (Fig. 3D–F).

### Inhibition of miR-34a-5p alleviated ATO-induced apoptosis in cardiomyocytes

**Fig. 4:**
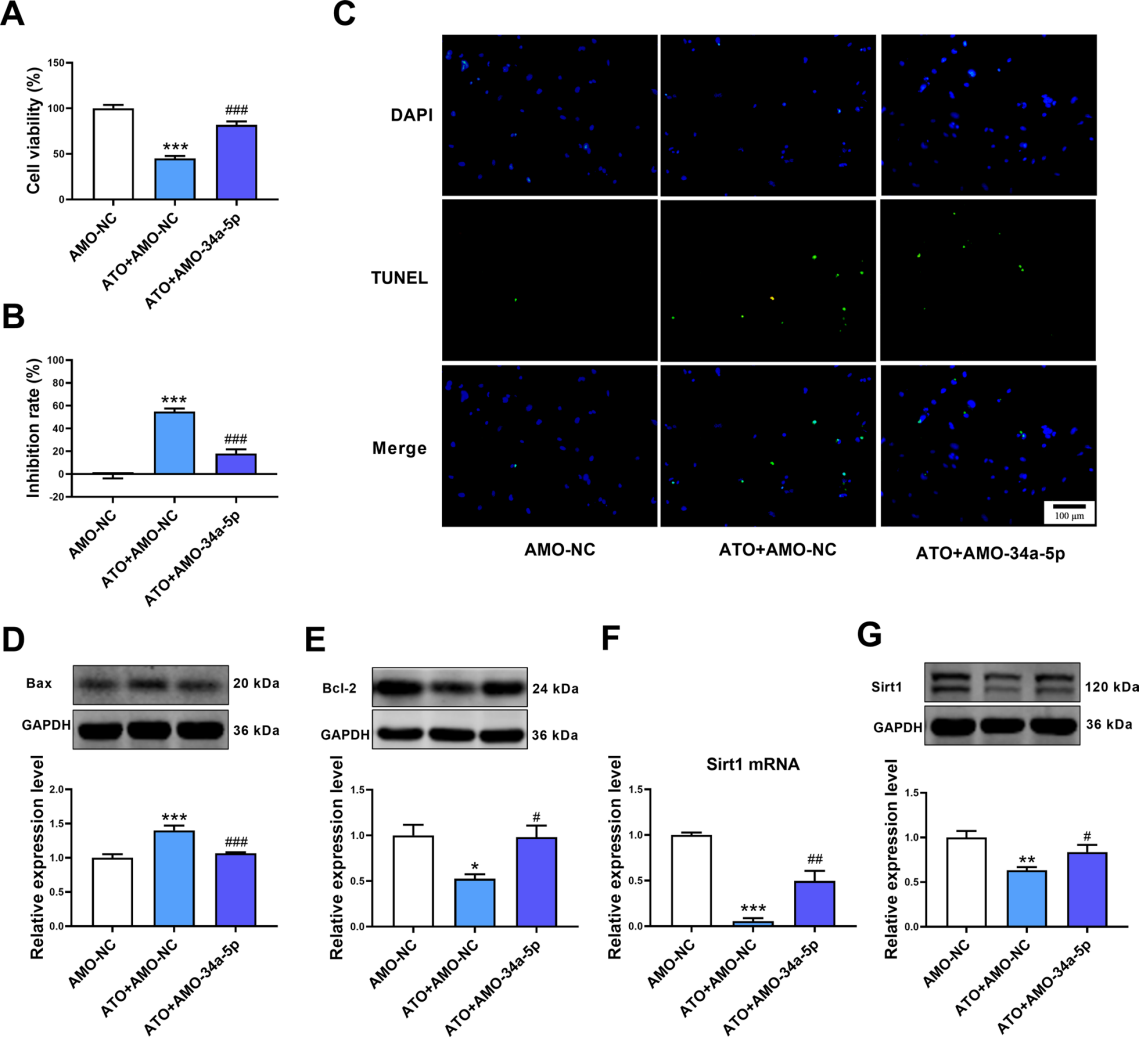
Inhibition of miR-34a-5p alleviated apoptosis of cardiomyocytes induced by ATO. **A** Inhibition of miR-34a-5p increased the viability of ATO-treated mouse cardiomyocytes. **B** Inhibition of miR-34a-5p decreased the inhibition rate of ATO-treated mouse cardiomyocytes. **C** Representative TUNEL staining images of mouse cardiomyocytes. Magnification: 200 × ; scale bar: 100 μm. **D** Downregulation of miR-34a-5p decreased Bax protein expression in ATO-treated mouse cardiomyocytes. **E** Downregulation of miR-34a-5p increased Bcl-2 protein expression in ATO-treated mouse cardiomyocytes. **F** Downregulation of miR-34a-5p increased Sirt1 mRNA expression in ATO-treated mouse cardiomyocytes. **G** Downregulation of miR-34a-5p increased Sirt1 protein expression in ATO-treated mouse cardiomyocytes. For **A**, **B**,** D**, **E**, **F** and **G**, one-way ANOVA F value = 66.59, 66.59, 17.14, 6.733, 48.78 and 7.761, respectively. *P < 0.05, **P < 0.01, ***P < 0.001 vs. AMO-NC group. ^#^P < 0.05, ^##^P < 0.01, ^###^P < 0.001 vs. ATO + AMO-NC group; **A**, **B**, n = 12; **C**–**G**, n = 3–6

The effect of miR-34a-5p on ATO-induced apoptosis in cardiomyocytes was measured. Downregulation of miR-34a-5p relieved the effect of ATO on cell viability and the inhibition rate in mouse cardiomyocytes (Fig. 4A, B). Moreover, transfection with AMO-34a-5p decreased the number of TUNEL-positive cells in ATO-treated cardiomyocytes (Fig. 4C), accompanied with decreased Bax and increased Bcl-2 protein expression (Fig. 4D, E). Simultaneously, transfection with AMO-34a-5p also alleviated the inhibitory effect of ATO on Sirt1 mRNA and protein expression in cardiomyocytes (Fig. 4F, G).

### The effect of lncRNA Kcnq1ot1 knockdown on miR-34a-5p and Sirt1 expression in mouse cardiomyocytes

**Fig. 5:**
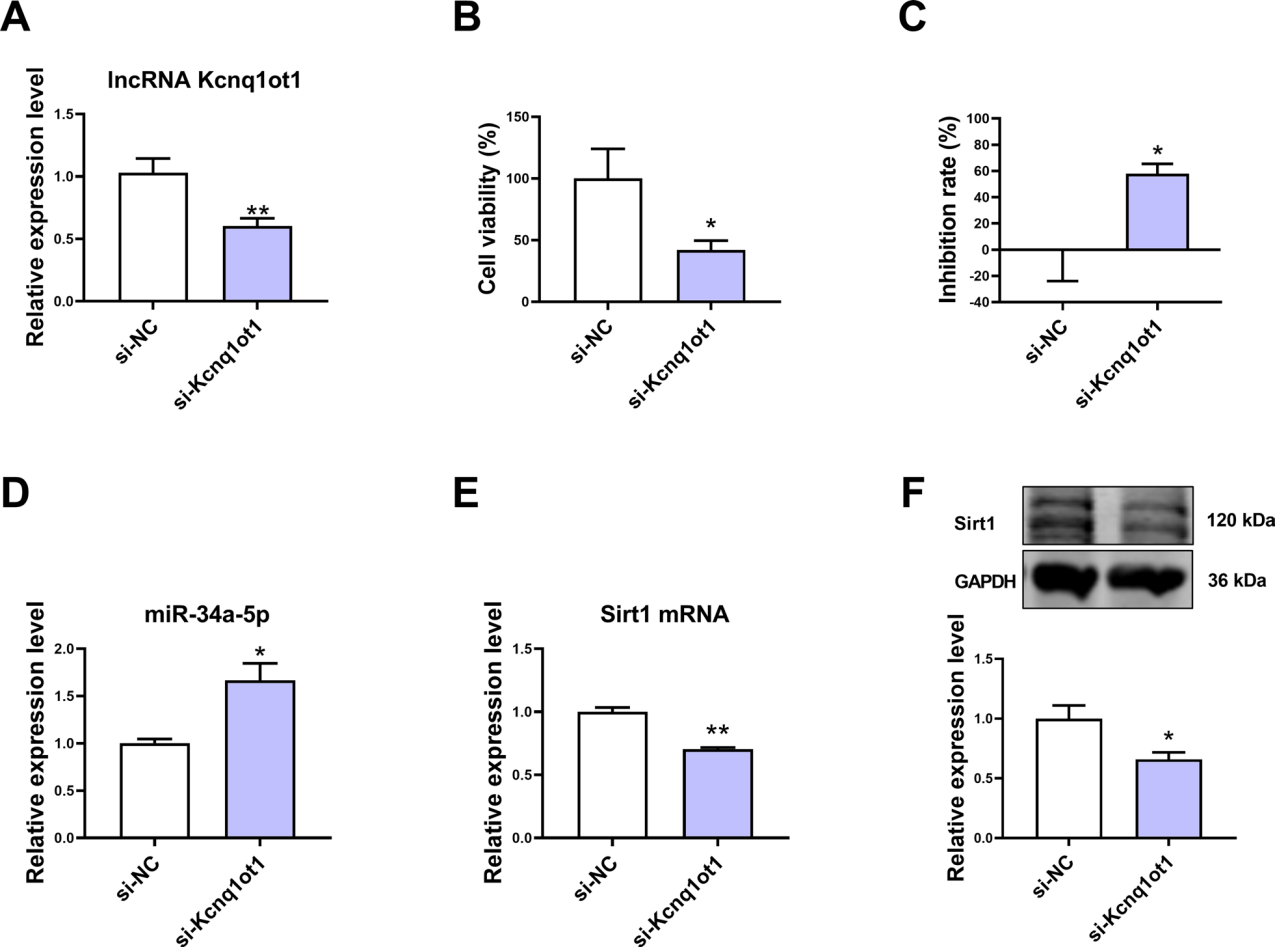
The effect of lncRNA Kcnq1ot1 knockdown on miR-34a-5p and Sirt1 expression in cardiomyocytes. **A** The expression of lncRNA Kcnq1ot1 was downregulated in si-Kcnq1ot1-transfected cardiomyocytes. **B** Knockdown of lncRNA Kcnq1ot1 decreased the viability of mouse cardiomyocytes. **C** Knockdown of lncRNA Kcnq1ot1 increased the inhibition rate of mouse cardiomyocytes. **D** Knockdown of lncRNA Kcnq1ot1 upregulated miR-34a-5p expression in cardiomyocytes. **E** Knockdown of lncRNA Kcnq1ot1 downregulated Sirt1 mRNA expression in cardiomyocytes. **F** Knockdown of lncRNA Kcnq1ot1 downregulated Sirt1 protein expression in cardiomyocytes. *P < 0.05, **P < 0.01 vs. si-NC group; n = 3–6

Transfection with si-Kcnq1ot1 downregulated the expression of lncRNA Kcnq1ot1 in cardiomyocytes (Fig. 5A). Knockdown of lncRNA Kcnq1ot1 expression reduced cell viability and increased the inhibition rate of cardiomyocytes (Fig. 5B, C). In addition, miR-34a-5p expression was upregulated and Sirt1 expression was downregulated after knockdown of lncRNA Kcnq1ot1 (Fig. 5D–F).

### Inhibition of miR-34a-5p relieved apoptosis of cardiomyocytes induced by lncRNA Kcnq1ot1 knockdown

**Fig. 6:**
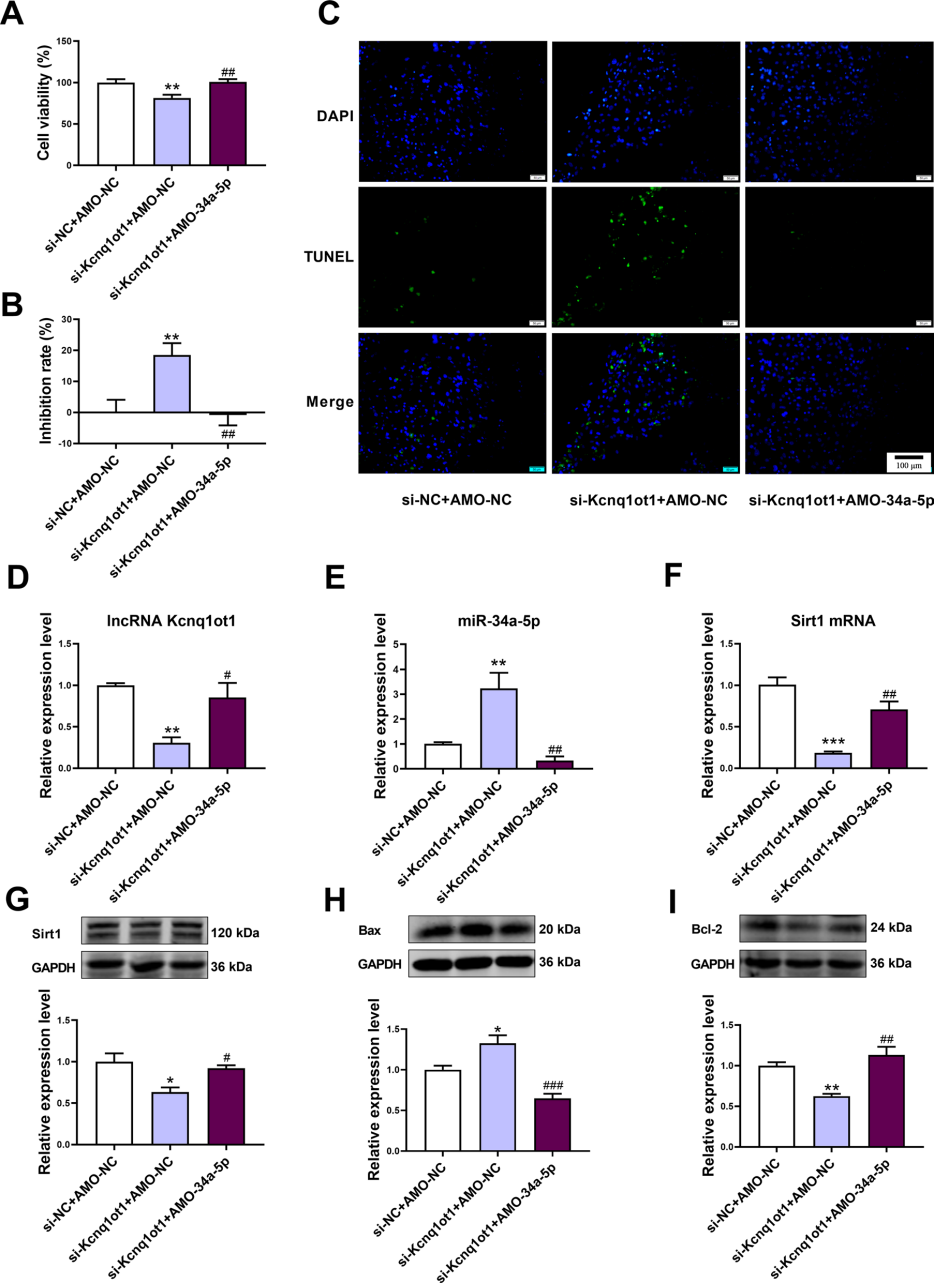
Inhibition of miR-34a-5p relieved the apoptosis of cardiomyocytes induced by lncRNA Kcnq1ot1 knockdown. **A** Inhibition of miR-34a-5p increased the viability of mouse cardiomyocytes with lncRNA Kcnq1ot1 knockdown. **B** Inhibition of miR-34a-5p decreased the inhibition rate of mouse cardiomyocytes with lncRNA Kcnq1ot1 knockdown. **C** Representative TUNEL staining images of mouse cardiomyocytes. Magnification: 200 × ; scale bar: 100 μm. **D** Inhibition of miR-34a-5p increased lncRNA Kcnq1ot1 expression in si-Kcnq1ot1-transfected mouse cardiomyocytes. **E** Transfection with si-Kcnq1ot1 increased miR-34a-5p expression, which was reversed by miR-34a-5p inhibition. **F** Inhibition of miR-34a-5p increased Sirt1 mRNA expression in si-Kcnq1ot1-transfected mouse cardiomyocytes. **G** Inhibition of miR-34a-5p increased Sirt1 protein expression in si-Kcnq1ot1-transfected mouse cardiomyocytes. **H** Inhibition of miR-34a-5p decreased Bax protein expression in si-Kcnq1ot1-transfected mouse cardiomyocytes. **I** Inhibition of miR-34a-5p increased Bcl-2 protein expression in si-Kcnq1ot1-transfected mouse cardiomyocytes. For **A**, **B**, and **D**–**I**, one-way ANOVA F value = 8.525, 8.525, 11.17, 16.29, 29.86, 7.684, 22.66 and 16.11, respectively. * P < 0.05, ** P < 0.01, ***P < 0.001 vs. si-NC + AMO-NC group. ^#^P < 0.05, ^##^P < 0.01, ^###^P < 0.001 vs. si-Kcnq1ot1 + AMO-NC group; for **A**, **B**, n = 10; for **C**–**I**, n = 3–4

The effect of lncRNA Kcnq1ot1 knockdown on cell viability and the inhibition rate was reversed by the inhibition of miR-34a-5p (Fig. 6A, B). Knockdown of lncRNA Kcnq1ot1 increased the number of TUNEL-positive cardiomyocytes, which was attenuated by transfection with AMO-34a-5p (Fig. 6C). In accordance with these results, the Bax protein expression was upregulated and Bcl-2 protein expression was downregulated after knockdown of lncRNA Kcnq1ot1 in cardiomyocytes, which was reversed by coadministration of AMO-34a-5p (Fig. 6H, I). In addition, downregulation of lncRNA Kcnq1ot1 upregulated miR-34a-5p and downregulated Sirt1 expression. However, transfection with AMO-34a-5p attenuated the effect of Kcnq1ot1 knockdown (Fig. 6D–G).

### Propranolol alleviated ATO-induced apoptosis in mouse cardiomyocytes and myocardial tissues

**Fig. 7:**
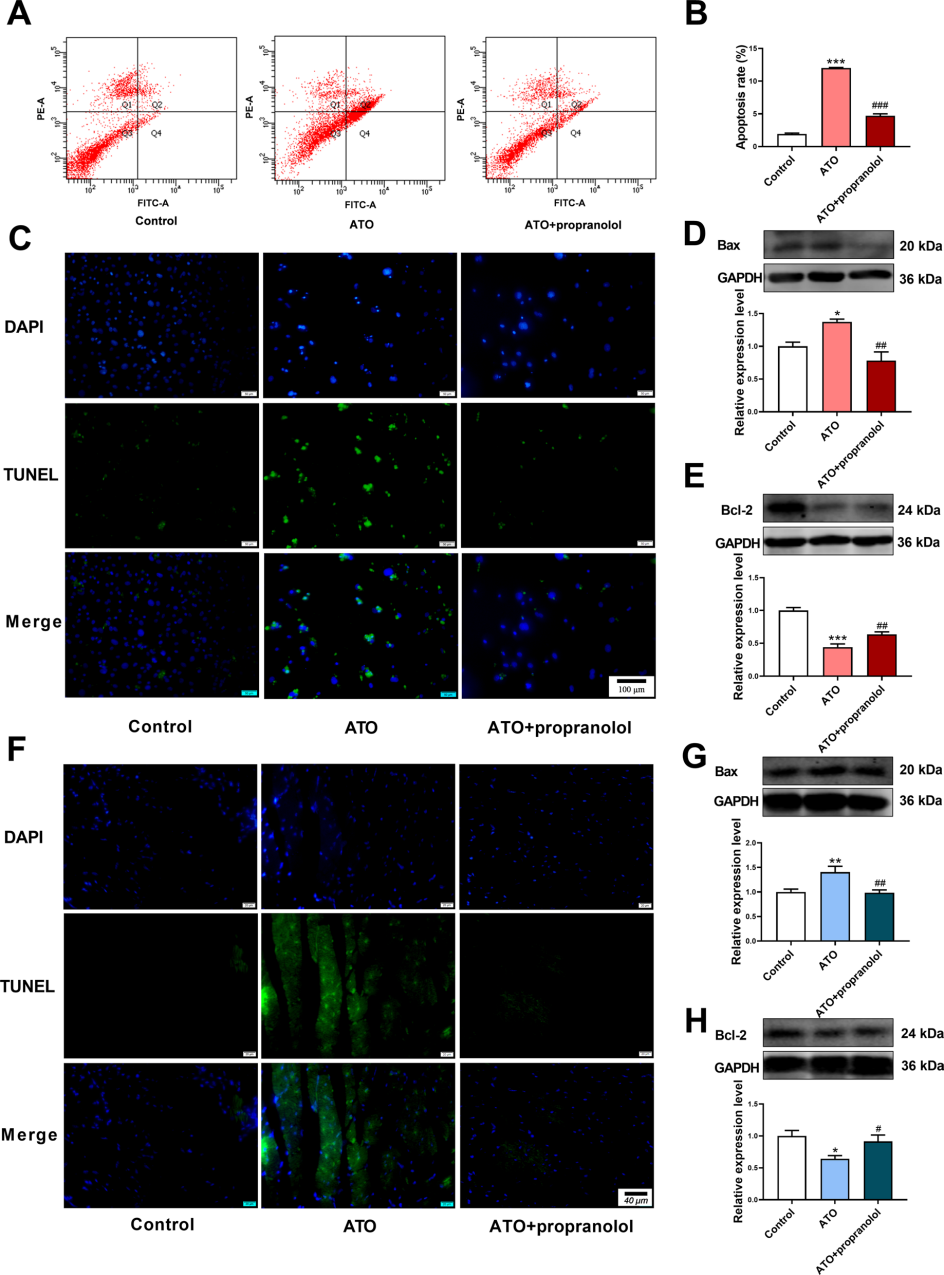
Propranolol alleviated ATO-induced apoptosis in mouse cardiomyocytes and myocardial tissues. **A** Representative flow cytometry images. **B** Statistical analysis of the flow cytometry results. **C** Representative TUNEL staining images of mouse cardiomyocytes. Magnification: 200 × ; scale bar: 100 μm. **D** Propranolol decreased Bax protein expression in ATO-treated mouse cardiomyocytes. **E** Propranolol increased Bcl-2 protein expression in ATO-treated mouse cardiomyocytes. **F** Representative TUNEL staining images of mouse myocardial tissues. Magnification: 400 × ; scale bar: 40 μm. **G** Propranolol decreased Bax protein expression in ATO-treated mouse myocardial tissues. **H** Propranolol increased Bcl-2 protein expression in ATO-treated mouse myocardial tissues. For **B**, **D**, **E**, **G** and **H**, one-way ANOVA F value = 709, 11.64, 41.51, 8.302 and 5.311, respectively. *P < 0.05, **P < 0.01, ***P < 0.001 vs. control group; ^#^P < 0.05, ^##^P < 0.01, ^###^P < 0.001 vs. ATO group; n = 3–6

Subsequently, we explored the therapeutic potential of the lncRNA Kcnq1ot1/miR-34a-5p/Sirt1 signaling pathway in ATO-induced cardiotoxicity. The protective effect of propranolol on ATO-induced apoptosis in cardiomyocytes was detected using flow cytometry assay (Fig. 7A, B). Propranolol decreased the number of TUNEL-positive cells in ATO-treated mouse cardiomyocytes (Fig. 7C), which also decreased Bax and increased Bcl-2 protein expression (Fig. 7D, E). Similar results were validated in vivo. Administration of propranolol decreased the number of TUNEL-positive cells in ATO-treated mouse myocardial tissues (Fig. 7F). Correspondingly, propranolol attenuated the alteration of Bax and Bcl-2 expression in ATO-treated mouse myocardial tissues (Fig. 7G, H).

### Propranolol attenuated the effect of ATO on the lncRNA Kcnq1ot1/miR-34a-5p/Sirt1 pathway in mouse cardiomyocytes and myocardial tissues

**Fig. 8:**
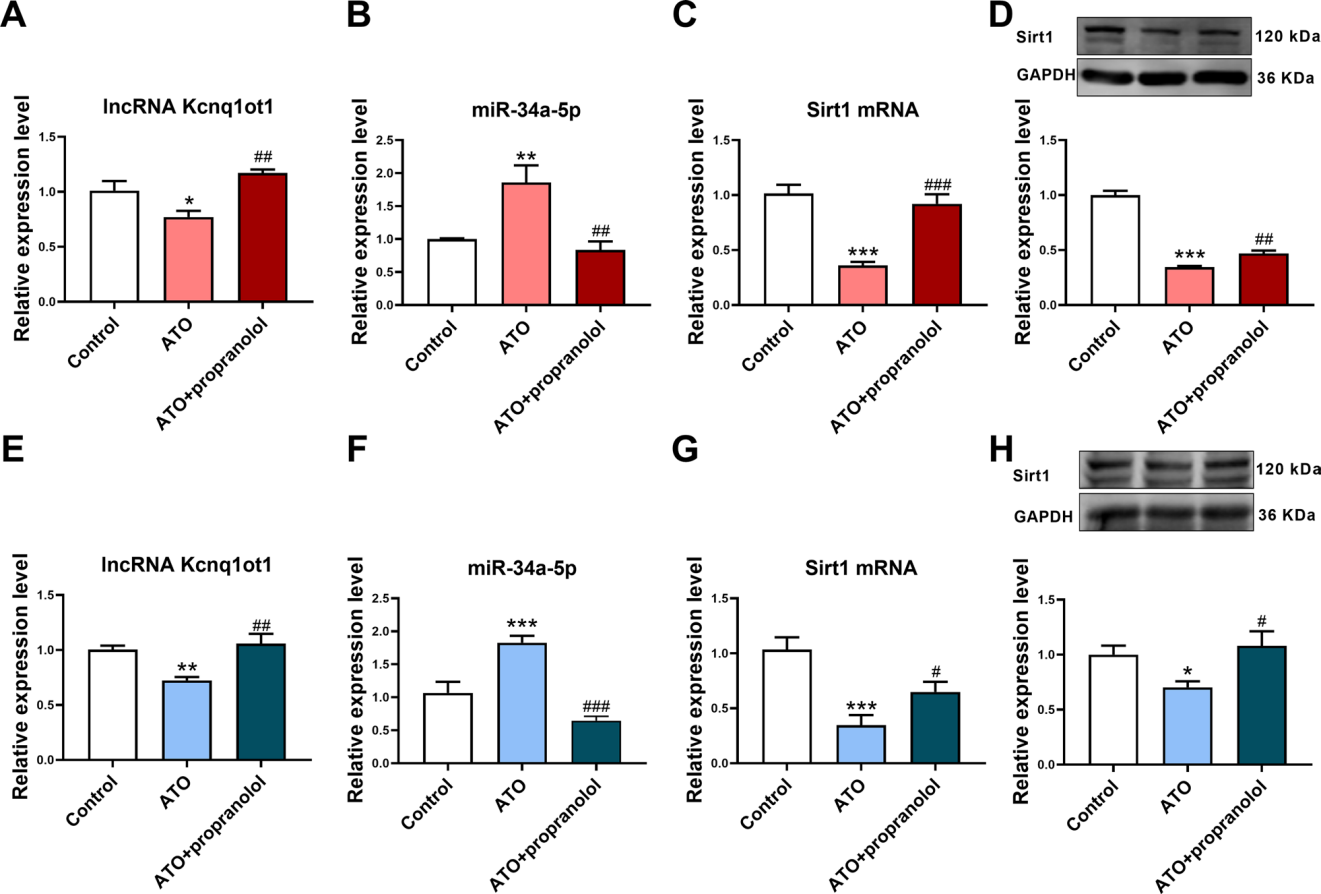
Propranolol attenuated the effect of ATO on the lncRNA Kcnq1ot1/miR-34a-5p/Sirt1 pathway in mouse cardiomyocytes and myocardial tissues. **A** Propranolol increased lncRNA Kcnq1ot1 expression in ATO-treated mouse cardiomyocytes. **B** Propranolol decreased miR-34a-5p expression in ATO-treated mouse cardiomyocytes. **C** Propranolol increased Sirt1 mRNA expression in ATO-treated mouse cardiomyocytes. **D** Propranolol increased Sirt1 protein expression in ATO-treated mouse cardiomyocytes. **E** Propranolol increased lncRNA Kcnq1ot1 expression in ATO-treated mouse myocardial tissues. **F** Propranolol decreased miR-34a-5p expression in ATO-treated mouse myocardial tissues. **G** Propranolol increased Sirt1 mRNA expression in ATO-treated mouse myocardial tissues. **H** Propranolol increased Sirt1 protein expression in ATO-treated mouse myocardial tissues. For **A–H**, one-way ANOVA F value = 10.17, 10.79, 25.16, 153.9, 9.598, 24.71, 12.15 and 4.356, respectively. *P < 0.05, **P < 0.01, ***P < 0.001 vs. control group; ^#^P < 0.05, ^##^P < 0.01, ^###^P < 0.001 vs. ATO group; n = 4–8

**Fig. 9:**
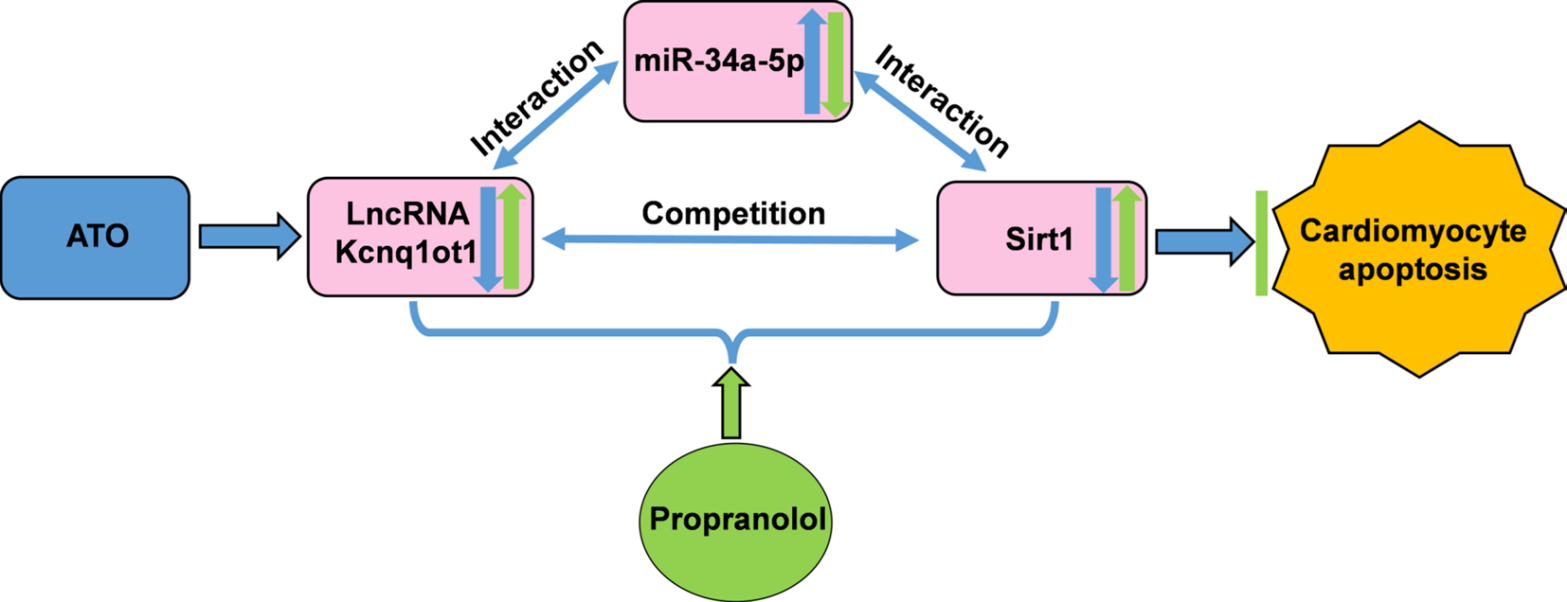
Schematic diagram of the mechanism of propranolol in the treatment for ATO-induced cardiotoxicity

Finally, the effect of propranolol on the lncRNA Kcnq1ot1/miR-34a-5p/Sirt1 pathway was detected. ATO decreased lncRNA Kcnq1ot1 and Sirt1 expression and increased miR-34a-5p expression in mouse cardiomyocytes and myocardial tissues, while propranolol attenuated the effect of ATO (Fig. 8A–H). These in vitro and in vivo results suggest that Propranolol can attenuate ATO-induced cardiotoxicity at least partially through the lncRNA Kcnq1ot1/miR-34a-5p/Sirt1 pathway (Fig. 9).

## Discussion

The cardiotoxicity of ATO is still a major problem in its clinical application. However, the involvement of lncRNAs in this process has not been fully clarified. Our previous work discovered that lncRNA Kcnq1ot1 is involved in the cardiotoxicity of ATO [26]. The present study further explored the role and underlying mechanism of lncRNA Kcnq1ot1 in ATO-induced cardiomyocyte apoptosis.

ATO at 10 mg/day (~ 0.15 mg/kg) is recommended for acute promyelocytic leukemia in the clinic. According to the conversion relationship between humans and mice, 10 mg/day for humans is approximately equal to 1.5 mg/kg for mice. In general, ATO is administered continuously for 2 weeks. Therefore, mice were administered ATO (1.5 mg/kg) for 2 weeks. For in vitro experiments, the dosage of ATO was screened by CCK-8 assay, and the relatively low and effective dose of ATO (5 μM) was selected in our experiments. In ATO-treated mouse cardiomyocytes and myocardial tissues, the number of TUNEL-positive cells was increased, which was associated with increased Bax and decreased Bcl-2 protein expression. These results were in accordance with previous findings that ATO can induce apoptosis of cardiomyocytes [24, 39, 40]. Then, the target of lncRNA Kcnq1ot1 was predicted based on the ceRNA theory. The results showed that lncRNA Kcnq1ot1 has binding sites with miR-34a-5p. Moreover, Sirt1 is a downstream target of miR-34a-5p. The dual-luciferase reporter assay results showed that mouse-derived miR-34a-5p has a direct binding site with lncRNA Kcnq1ot1. For humans, the direct binding site between lncRNA Kcnq1ot1 and miR-34a-5p was validated using dual-luciferase reporter assay [41]. In addition, the direct binding site between miR-34a-5p and Sirt1 has previously been validated using dual-luciferase reporter assay [42, 43]. Therefore, the lncRNA Kcnq1ot1/miR-34a-5p/Sirt1 pathway may be involved in the cardiotoxicity of ATO. The expression of lncRNA Kcnq1ot1 and Sirt1 was downregulated and that of miR-34a-5p was upregulated in ATO-treated mouse myocardial tissues and cardiomyocytes, which is consistent with ceRNA theory.

MiR-34a-5p has been verified to be increased in cardiomyocytes undergoing apoptosis induced by different factors, such as ischemia, hypoxia, and doxorubicin. This enhanced expression can aggravate cardiomyocyte apoptosis, while inhibition of miR-34a-5p can protect against cardiomyocyte apoptosis [44–47]. In our study, miR-34a-5p was overexpressed or inhibited in cardiomyocytes to observe its effect on Sirt1 expression. The upregulation of miR-34a-5p inhibited Sirt1 expression, while the downregulation of miR-34a-5p increased Sirt1 expression. The involvement of the miR-34a-5p/Sirt1 pathway was detected by inhibition of miR-34a-5p in ATO-treated cardiomyocytes. The inhibition of miR-34a-5p alleviated ATO-induced apoptosis in mouse cardiomyocytes and attenuated the inhibitory effect of ATO on Sirt1 expression. Sirt1 is an NAD + -dependent deacetylase that is involved in the regulation of cellular senescence and apoptosis[48–50]. Enhanced Sirt1 expression exerts a protective effect on cardiomyocytes [50, 51]. In addition, the miR-34a-5p/Sirt1 pathway contributes to doxorubicin-induced cardiomyocyte apoptosis [47].

The effect of lncRNA Kcnq1ot1 on cardiomyocytes and the miR-34a-5p/Sirt1 pathway was then detected. The results showed that knockdown of lncRNA Kcnq1ot1 promoted apoptosis of cardiomyocytes. In addition, miR-34a-5p was upregulated and Sirt1 was downregulated after knockdown of lncRNA Kcnq1ot1 in cardiomyocytes. While, administration of AMO-34a-5p attenuated the effect of lncRNA Kcnq1ot1 knockdown. The above findings suggest that the lncRNA Kcnq1ot1/miR-34a-5p/Sirt1 pathway is involved in ATO-induced cardiotoxicity.

Subsequently, we explored the potential of the lncRNA Kcnq1ot1/miR-34a-5p/Sirt1 pathway as a therapeutic target for ATO-induced cardiotoxicity. Cardioprotective drugs may alleviate ATO-induced cardiotoxicity through the lncRNA Kcnq1ot1/miR-34a-5p/Sirt1 pathway. The beta-blocker propranolol is a widely used cardioprotective agent [52]. We explored the effect of propranolol on ATO-induced cardiotoxicity. Coadministration of propranolol alleviated ATO-induced apoptosis in mouse myocardial tissues and cardiomyocytes. Similarly, propranolol has also been shown to alleviate clozapine-induced cardiac oxidative stress injury and cardiomyocyte apoptosis[53]. Finally, we detected the effect of propranolol on the lncRNA Kcnq1ot1/miR-34a-5p/Sirt1 pathway. The results showed that propranolol increased lncRNA Kcnq1ot1 and Sirt1 expression, and decreased miR-34a-5p expression in ATO-treated mouse cardiomyocytes and myocardial tissues. These findings suggest that propranolol alleviated ATO-induced cardiomyocyte apoptosis both in vitro and in vivo, which at least partially through the lncRNA Kcnq1ot1/miR-34a-5p/Sirt1 pathway.

## Conclusions

In conclusion, the lncRNA Kcnq1ot1/miR-34a-5p/Sirt1 pathway is involved in ATO-induced cardiotoxicity. Propranolol can attenuate ATO-induced cardiotoxicity at least partially through the lncRNA Kcnq1ot1/miR-34a-5p/Sirt1 pathway. Combined administration with propranolol may be a new strategy for alleviating the cardiotoxicity of ATO. This study revealed a new mechanism of ATO-induced cardiotoxicity and a molecular basis of combined application of ATO and propranolol, which provides new insight into the study and rational use of ATO in the clinic.

## Data Availability

The primary data of the current study are available from the corresponding author on reasonable request.

## Abbreviations

ATO: Arsenic trioxide
AMO-34a-5p: Antisense morpholino oligonucleotide targeting miR-34a-5p
Bcl-2: B-cell lymphoma-2
Bax: Bcl-2-associated X Protein
ceRNA: Competing endogenous RNA
DMEM: Dulbecco's modified Eagle's medium
ECG: Electrocardiogram
FBS: Fetal bovine serum
lncRNA: Long noncoding RNA
miRNA: MicroRNA
PBS: Phosphate buffered saline
si-Kcnq1ot1: Small interfering RNA targeting lncRNA Kcnq1ot1
Sirt1: Silencing information regulator 2 related enzyme 1
