# Correction: The involvement and therapeutic potential of lncRNA Kcnq1ot1/miR-34a-5p/Sirt1 pathway in arsenic trioxide-induced cardiotoxicity

**DOI:** 10.1186/s12967-023-03982-2

**Published:** 2023-03-03

**Authors:** Xiuyun Shen, Fengnan Zhi, Chunpeng Shi, Jincheng Xu, Yuqiu Chao, Juan Xu, Yanan Jiang, Yunlong Bai, Baofeng Yang

**Affiliations:** 1 Department of Pharmacology (State-Province Key Laboratories of Biomedicine-Pharmaceutics of China, Key Laboratory of Cardiovascular Research, Ministry of Education), College of Pharmacy, Harbin Medical University, Harbin, China; 2 Translational Medicine Research and Cooperation Center of Northern China, Heilongjiang Academy of Medical Sciences, Harbin, China; 3 College of Bioinformatics Science and Technology, Harbin Medical University, Harbin, China; 4 Research Unit of Noninfectious Chronic Diseases in Frigid Zone, Chinese Academy of Medical Sciences (2019RU070), Harbin, China

**Correction: Journal of Translational Medicine (2023) 21:52** 10.1186/s12967-023-03895-0

Following publication of the original article [1], we have been notified that the sequence of authors was incorrectly published in the article. The correct sequence of authors in this correction:

Xiuyun Shen^1†^, Fengnan Zhi^1†^, Chunpeng Shi^1^, Jincheng Xu^1^, Yuqiu Chao^1^, Juan Xu^3^, Yanan Jiang^1,2*^, Yunlong Bai^1,2*^ and Baofeng Yang^1,2,4^
